# Circulating extracellular vesicles activate the pyroptosis pathway in the brain following ventilation-induced lung injury

**DOI:** 10.1186/s12974-021-02364-z

**Published:** 2021-12-29

**Authors:** Laura Chavez, Julia Meguro, Shaoyi Chen, Vanessa Nunes de Paiva, Ronald Zambrano, Julia M. Eterno, Rahul Kumar, Matthew R. Duncan, Merline Benny, Karen C. Young, W. Dalton Dietrich, Roberta Brambilla, Shu Wu, Augusto F. Schmidt

**Affiliations:** 1 Department of Pediatrics, University of Miami Miller School of Medicine, 1611 NW 12th Ave, Miami, FL 33136 USA; 2 The Miami Project To Cure Paralysis, Department of Neurological Surgery, University of Miami Miller School of Medicine, Miami, FL USA

**Keywords:** Prematurity, Mechanical ventilation, Lung injury, Brain injury, Gasdermin D, Pyroptosis

## Abstract

**Background:**

Mechanical ventilation of preterm newborns causes lung injury and is associated with poor neurodevelopmental outcomes. However, the mechanistic links between ventilation-induced lung injury (VILI) and brain injury is not well defined. Since circulating extracellular vesicles (EVs) are known to link distant organs by transferring their cargos, we hypothesized that EVs mediate inflammatory brain injury associated with VILI.

**Methods:**

Neonatal rats were mechanically ventilated with low (10 mL/kg) or high (25 mL/kg) tidal volume for 1 h on post-natal day 7 followed by recovery for 2 weeks. Exosomes were isolated from the plasma of these rats and adoptively transferred into normal newborn rats. We assessed the effect of mechanical ventilation or exosome transfer on brain inflammation and activation of the pyroptosis pathway by western blot and histology.

**Results:**

Injurious mechanical ventilation induced similar markers of inflammation and pyroptosis, such as increased IL-1β and activated caspase-1/gasdermin D (GSDMD) in both lung and brain, in addition to inducing microglial activation and cell death in the brain. Isolated EVs were enriched for the exosomal markers CD9 and CD81, suggesting enrichment for exosomes. EVs isolated from neonatal rats with VILI had increased caspase-1 but not GSDMD. Adoptive transfer of these EVs led to neuroinflammation with microglial activation and activation of caspase-1 and GSDMD in the brain similar to that observed in neonatal rats that were mechanically ventilated.

**Conclusions:**

These findings suggest that circulating EVs can contribute to the brain injury and poor neurodevelopmental outcomes in preterm infants with VILI through activation of GSDMD.

**Supplementary Information:**

The online version contains supplementary material available at 10.1186/s12974-021-02364-z.

## Introduction

Mechanical ventilation is a life-saving therapy for extremely preterm infants, but it is associated with both lung and brain injury in these infants [1, 2]. While ventilation strategies have evolved to minimize lung injury from mechanical ventilation by close monitoring and limiting delivered tidal volumes, during neonatal resuscitation positive pressure ventilation is often uncontrolled and newborns frequently receive large tidal volume breaths, sometimes up to four times the physiological tidal volume [3]. In preterm lambs as few as 15 min of large tidal volume (Vt) ventilation can induce lung inflammation and systemic acute phase response [4]. In the short term, mechanical ventilation can lead to fluctuations in cerebral blood flow and induce brain injury and inflammation acutely [5, 6]. In the long term, use of mechanical ventilation is an important contributor to the development of bronchopulmonary dysplasia, which by itself is an independent risk factor for neurodevelopmental impairment in preterm infants [7, 8]. However, a link between ventilation-induced lung injury (VILI) and brain injury has not been established.

Extracellular vesicles (EVs) are involved in intercellular and interorgan communication in diverse physiological and pathological processes including inflammatory states [9, 10]. EVs carry cytokines, caspases, and miRNAs that can signal inflammation in the target cell [11]. In traumatic brain injury, EVs have been shown to contain inflammasome proteins, whose activation can lead to acute lung injury [12]. More recently, we have shown that circulating EVs from newborn rats exposed to hyperoxia contain inflammasome proteins that can induce lung and brain inflammation when transfected into normal animals [13]. These exosomes also contained gasdermin D (GSDMD)—a molecule downstream from caspase-1—that is the effector of pyroptosis. The procaspase-1 protein (30–48 kDa) is cleaved upon activation of the inflammasome pathway releasing a 20 kDa active fragment, this active fragment is responsible for cleavage of pro-IL-1β releasing IL-1β, and cleavage of the 53 kDa GSMD releasing the active 30 kDa fragment that can oligomerize and form the pyroptotic pore in the cell membrane [14].

Based on these observations, we hypothesized that VILI could cause the release of EVs containing pyroptosis mediators into the systemic circulation and that these EVs could cross the blood brain barrier to induce inflammation and pyroptosis in the brain.

## Methods

### Animal model of neonatal mechanical ventilation

Animal experiments were approved by the Institutional Animal Care and Use Committee at the University of Miami Miller School of Medicine (19-011). Pregnant Sprague–Dawley rats were purchased from Charles River Laboratories (Wilmington, MA, USA). Each pregnant animal was housed in a single cage with water and food ad libitum under 12 h night and day cycles under controlled temperature and humidity. Newborn Sprague–Dawley rats on postnatal day (P) 7 were anesthetized with a mixture of Ketamine and Xylazine (80 mg/kg and 10 mg/kg, respectively) by intraperitoneal injection, orally intubated with a 22G intravenous catheter, connected to a small animal mechanical ventilator (MiniVent, Harvard Apparatus, Cambridge, MA, USA) and ventilated for 30–60 min with tidal volume of 10 mL/kg (low Vt) or 25 mL/kg (high Vt). The respiratory rate was set to 150 in the low Vt group and 80 in the high Vt group to maintain a similar minute ventilation between the ventilated groups. During mechanical ventilation, the animal was continuously monitored for heart rate, respiratory rate, oxygen saturation, and body temperature with a small animal monitoring station (Harvard Apparatus, Cambridge, MA, USA). Animals received blended supplemental oxygen as needed to maintain blood oxygen saturation above 85%. Control animals were not ventilated. At the end of ventilation, we progressively weaned the ventilator settings to allow return of spontaneous respirations and then rats were returned to the nursing dam for growth under normal conditions. On P21 (2 weeks after mechanical ventilation), rats were euthanized and plasma, lung, and brain tissue were sampled.

### Antibodies

Antibodies information and dilutions used for different assays are shown in Additional file 1: Tables S1 and S2. The anti-caspase-1 and anti-GSDMD antibodies detect both the full protein and the cleaved active fragment, which can be discerned in Western blot by the differences in molecular weight.

### Histological analysis

Lungs were inflation-fixed with 4% paraformaldehyde at 20 cmH_2_O via a tracheal cannula for 5 min and then fixed overnight. Following fixation samples were embedded in paraffin, and sectioned. Hematoxylin/eosin staining was performed and the mean linear intercept (MLI) was calculated as previously described [15]. Brain were fixed in 4% paraformaldehyde without perfusion. Coronal section of 10 µm were obtained and sections from similar areas were selected for staining based on the position and visible structures on adjacent section stained for hematoxylin–eosin.

For immunohistochemical staining, paraffin embedded brain sections of 10 µm were deparaffinized and rehydrated before heat-assisted antigen retrieval in citric acid buffer at pH 6.0. Endogenous peroxidase activity was blocked with CH_3_OH/H_2_O_2_ treatment and non-specific binding sites were blocked with 4% BSA in PBS. Sections were incubated overnight at 4 °C with the primary antibody diluted in 4% BSA in PBS. Sections were then washed and incubated with the appropriate species-specific secondary antibody diluted 1:200 in 4% BSA for 2 h at room temperature. After further washing, antigen:antibody complexes were visualized using a Vectastain ABC peroxidase kit (Vector Laboratories Inc., Burlingame, CA, USA). Antigen detection was enhanced with 3,3′-diaminobenzidine tetrahydrochloride. Slides were then counterstained with Harris hematoxylin.

Analysis of immunohistochemical section for IBA-1 were counted by 2 independent researchers blinded to group assignment. Qualitative analysis of the positive cells was performed by these investigators based on size of the soma and branching complexity.

To investigate the presence of cell death we performed terminal deoxynucleotidyl transferase-mediated deoxyuridine triphosphate nick end labeling (TUNEL) assays with a commercial kit according to the manufacturer’s instructions (Click-iT TUNEL Assay, ThermoFisher, Waltham, MA, USA).

### EV isolation, characterization, and adoptive transfer

EVs from an equal volume of plasma from control and ventilated rats were isolated 2 weeks after mechanical ventilation using the Total Exosome Isolation Kit (ThermoFisher, Waltham, MA, USA) per manufacturer’s instructions. In brief the reagent was added to plasma samples and incubated for 30 min at 4 ºC followed by centrifugation at 10,000×*g* for 5 min at room temperature. The pellets were re-suspended in PBS for downstream analyses. Plasma samples did not undergo sonication or treatment with protease/phosphatase inhibitors. A volume of 4 µL from each EV sample was analyzed for particle number and size distribution by nanoparticle tracking analysis using the Nanosight NS300 system (Malvern Instruments, Malvern, UK) [12]. EV tagging for imaging experiments was performed using the ExoGlow-Vivo EV Labeling Kit (Systems Biosciences, Palo Alto, CA, USA) followed by in vivo whole body imaging using the In Vivo Imaging System (IVIS, PerkinElmer, Hopkinton, MA, USA), followed by dissection and ex vivo imaging in the same system. Adoptive transfer of exosomes was performed in normal rats on P8 and P14 via tail vein injection of a protein-based dose of EVs of 50 µg per dose animal.

### Western blot analyses

Protein concentrations of tissue homogenates or EVs were measured by BCA protein assay using a commercial kit (Pierce Biotechnology Inc., Rockford, IL, USA). Total proteins (20 µg/sample) were fractionated by SDS-PAGE on 4–12% Tris–glycine precast gradient gels (ThermoFisher, Waltham, MA, USA) and then transferred to nitrocellulose membranes (Amersham, Piscataway, NJ, USA). The membranes were incubated overnight at 4 °C with the respective primary antibodies and then incubated for 1 h at room temperature with HRP-conjugated secondary antibodies. Antibody bound proteins were detected using ECL chemiluminescence methodology (Amersham, Piscataway, NJ, USA). The intensities of protein bands were quantified by ImageJ [16]. Band density was then corrected for beta-actin band density in the same lane. Values were then divided for the average control value to represent fold change relative to control.

### Cytokine/chemokine assay

Rat cytokine/chemokine concentrations in plasma, lung homogenates, and corpus callosum homogenates was determined by rat cytokine array/chemokines array-27 (Eve Technologies, Calgary, Canada). Blood was obtained by cardiac puncture at the time of euthanasia, placed on heparin tubes and vortexed for plasma extraction. Flash frozen lung and brain tissue were homogenized in RIPA buffer (Santa Cruz Biotechnology, catalog # sc-24948) and centrifuged at 12,000 rpm for 20 min at 4 ºC. The supernatant was transferred to a new tube and protein concentration was measured by BCA protein assay (Thermo Scientific, catalog # 23228 and 1859078). Samples were than diluted for a target protein concentration of 3–4 mg/mL. Values for samples with signal outside the curve were calculated when feasible by the model. Lower limit of detection for plasma and tissue samples are shown in Additional file 1: Table S3.

### Data analysis

Statistical analyses was performed using GraphPad Prism with ANOVA for parametric or Kruskal–Wallis for nonparametric test performed on each outcome related to GSDMD, caspase 1 expression, brain inflammatory markers, and cellular death. Decision for use of parametric or nonparametric test was based both on normality and lognormality distribution using the Shapiro–Wilk test as well as based on visual analysis for data distribution, outliers, and prior experience with the experiments in this model. Specific tests used for each analyses are described in the results. For ventilation-induced lung and brain injury experiments a total of 10 animals were created per group. For the analysis of lung structure and MLI we excluded the animals that had inconsistent inflation fixation which would bias the results. Animals used in the MLI analysis were given preference for downstream analyses, including exosome analyses and adoption transfer experiments. For the TUNEL assays due to low number of TUNEL positive cells present in the brain tissue overall across all groups all 10 animal were included in the analysis.

## Results

### VILI activates caspase-1/GSDMD in the lung

**Fig. 1:**
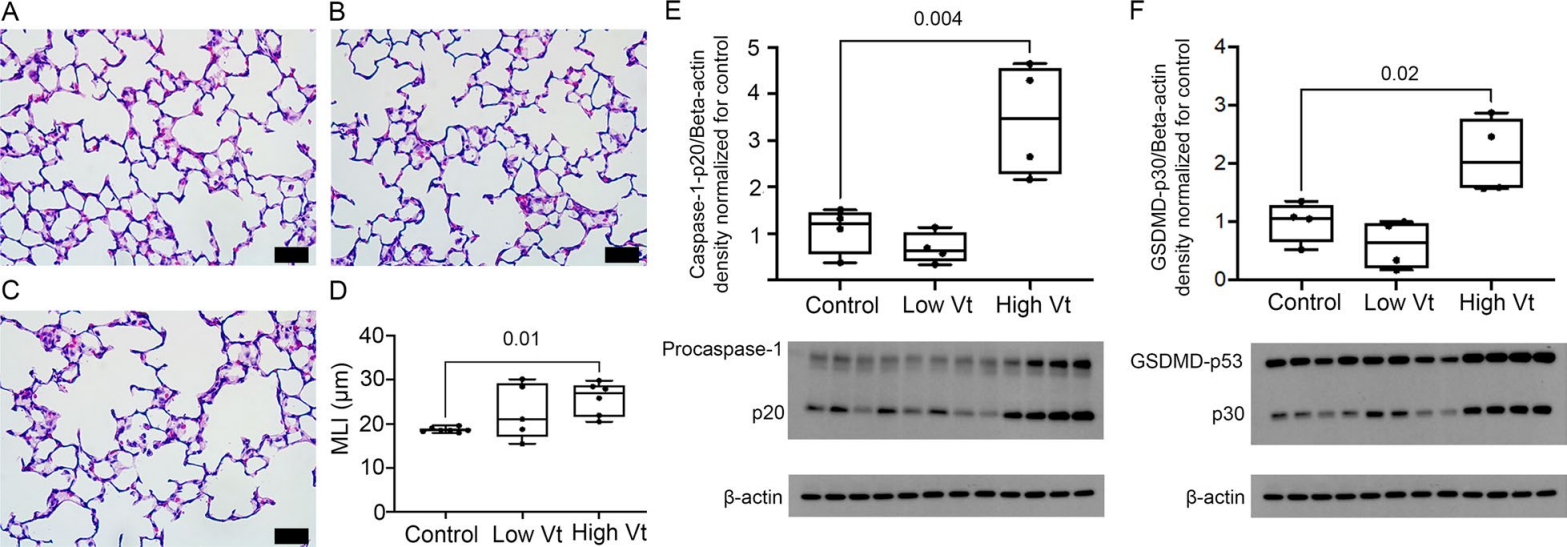
VILI causes alveolar simplification and activates caspase-1 and GSDMD in the neonatal lung. **A–D** Photomicrographs (20×; bars = 50 µm) of H&E-stained lung sections from rats on P21 of representative control animals (**A**) and rats mechanically ventilated with low (**B**) or high Vt (**C**) showing alveolar simplification and increased mean linear intercept (**D**) in animals exposed to high Vt mechanical ventilation. *N* = 6 controls, 5 low Vt, and 6 High Vt. **E–F** Western blots showing increased active caspase-1 (p20 fragment) (**E**) and active GSDMD (p30 fragment) (**F**) in lung homogenates 2 weeks after injurious mechanical ventilation with high Vt. *N* = 4 controls, 4 low Vt, and 4 High Vt. Data are presented as box plots showing the median, 25th to 75th percentile, and minimal to maximal value normalized to control. Data was analyzed by ANOVA with Dunnett’s post-test, *p* values < 0.05 are shown on bars

To understand the role of VILI on neonatal brain inflammation and injury we ventilated newborn rats on postnatal days 7 with low (10 mL/kg) or high Vt (25 mL/kg) for 1 h. After ventilation, animals were extubated and returned to the nursing dam. Animal data are shown in Table 1. To verify that our model caused ventilation-induced lung injury we assessed lung alveolarization in histological lung sections by measuring the MLI. Neonatal rats ventilated with high tidal volume had increased MLI compared to unventilated animals showing alveolar simplification at 2 week post-ventilation (Fig. 1A–D). We then assessed whether ventilation induced lung injury also induced the expression and activation of caspase-1 and GSDMD in the lung. High Vt mechanical ventilation increased total and active caspase-1 (Fig. 1E) and GSDMD at 2 weeks (Fig. 1F) after mechanical ventilation, suggesting activation of pyroptosis in VILI.

**Table 1:** Animal data

	Control	Low Vt	High Vt	EV-injected		
				Control EV	Low Vt EV	High Vt EV
*n*	10	10	10	5	5	5
Age at ventilation	P7	P7	P7	n/a	n/a	n/a
Age at EV injection #1/#2	n/a	n/a	n/a	P8/P14	P8/P14	P8/P14
Age at sampling	P21	P21	P21	P21	P21	P21
Weight (g)	70 ± 4.8	71 ± 6.8	70 ± 8.8	74 ± 3.7	77 ± 3.7	73 ± 3.5
Sex (M/F)	5/5	5/5	6/4	3/2	2/3	2/3

### VILI causes brain inflammation and cell death

**Fig. 2:**
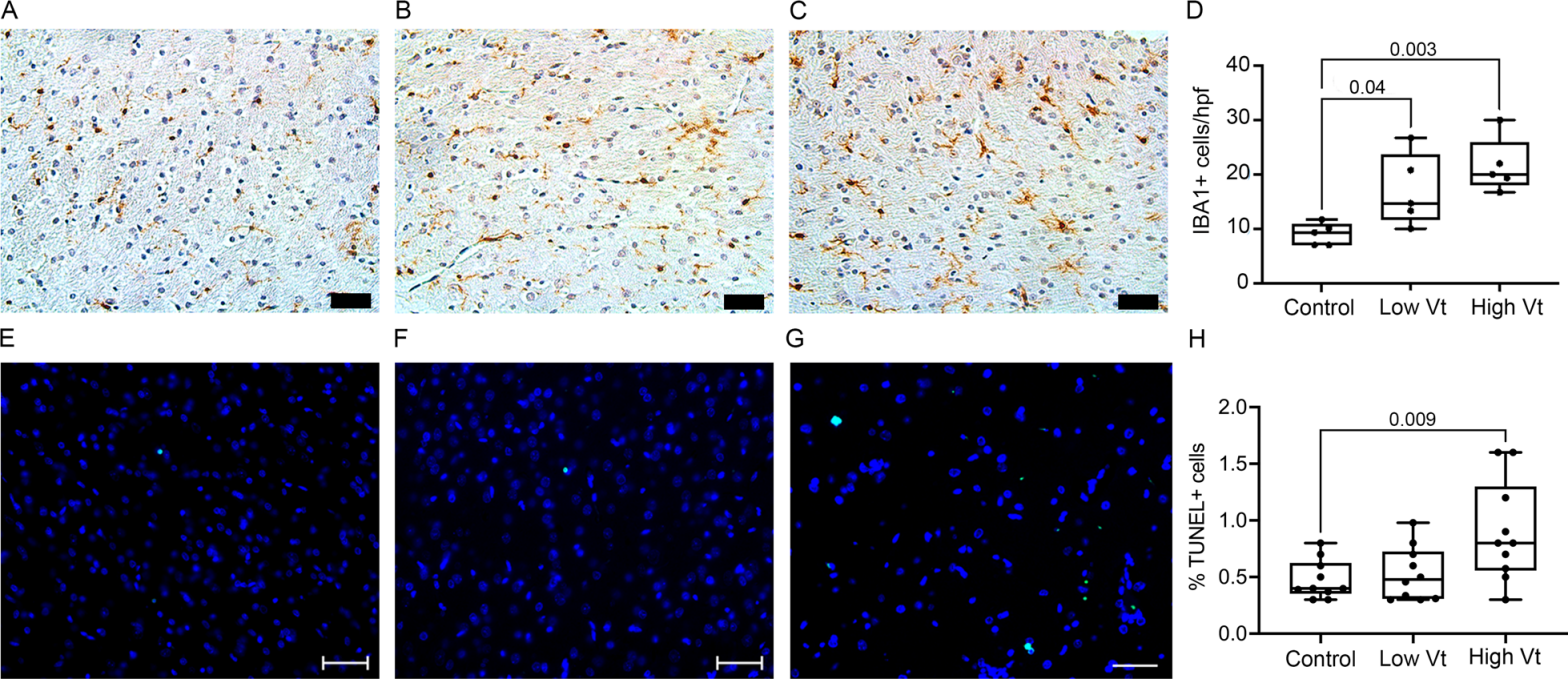
Injurious mechanical ventilation causes brain inflammation and cell death. **A–D** Microglia count and activation. Photomicrographs (20×; bars = 50 µm) of representative brain section immunostained for the microglial marker IBA-1 showing the corpus callosum from controls (**A**), low Vt (**B**), and high Vt (**C**) animals. There was microglial count (**D**) and morphological changes of the microglia consistent with microglial activation (inserts) with increased soma and thickened ramifications in ventilated animals. *N* = 5 animals/group. **E–H** TUNEL assay for assessment of cellular death. Photomicrographs of fluorescent stained brain sections for TUNEL assay of the corpus callosum from controls (**E**), low Vt (**F**), and high Vt (**G**) animals. There was an increased proportion of TUNEL + cells in animals ventilated with high Vt (**H**). *N* = 10 animals per group. Data are presented as box plots showing the median, 25th to 75th percentile, and minimal to maximal value. Data was analyzed by ANOVA with Dunnett’s post-test, *p* values < 0.05 are shown on bars. *Hpf* high-power field

We then assessed the effects of VILI on brain inflammation through microglial activation by IBA-1 immunostaining in the brain of controls and ventilated rats 2 weeks after mechanical ventilation. Rats ventilated with low or high Vt in the neonatal period showed an increased number of microglia in the corpus callosum compared to controls (Fig. 2A–D). The microglia in the animals with VILI also showed a more active phenotype with larger soma and thicker ramifications. This microglial activation was associated with increased cell death in the white matter of rats ventilation with high tidal volume, as identified by TUNEL assay (Fig. 2E–H), suggesting neonatal VILI leads to microglial activation and cell death in the corpus callosum and subcortical white matter (Fig. 2E–H).

**Fig. 3:**
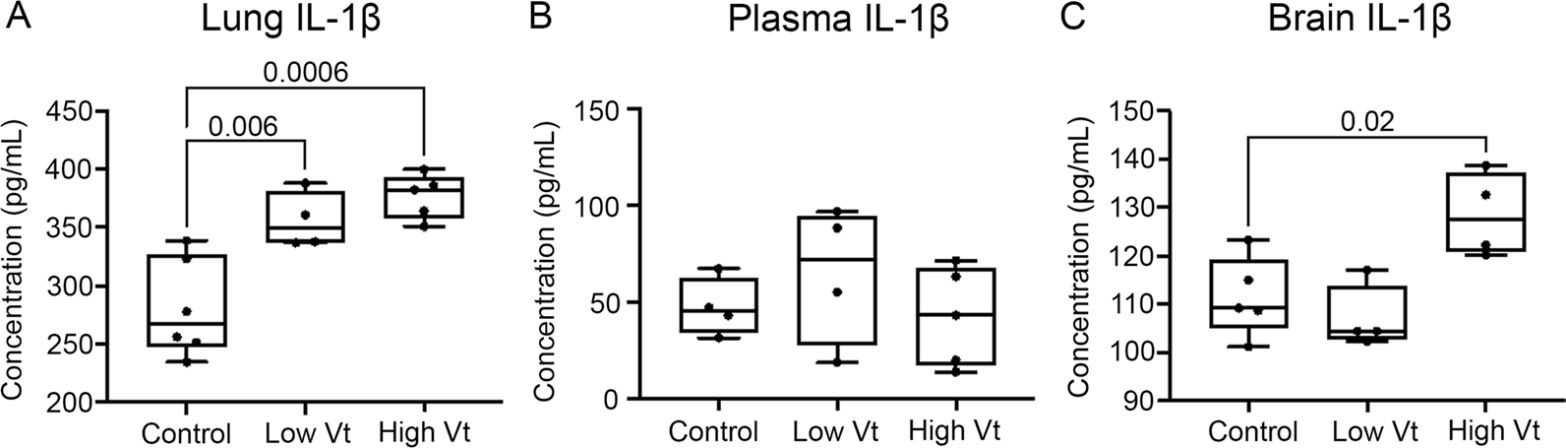
VILI increases IL-1β in the lung and corpus callosum but not in the plasma at 2 week post injury. Multiplex ELISA shows increased IL-1β in the lung (**A**) and in the corpus callosum (**C**), but not in the plasma (**B**) of animals with VILI. *N* = 6 controls, 4 low Vt, and 5 high Vt. Data are presented as box plots showing the median, 25th to 75th percentile, and minimal to maximal value. Data was analyzed with the nonparametric Kruskal–Wallis test with Dunn’s post-test for multiple comparison, *p* values < 0.05 are shown on bars

To determine whether lung derived cytokines from VILI animals could be contributing to the continued microglial activation and cell death in the brain at 2 weeks after mechanical ventilation, we measured cytokine and chemokine concentrations in the lung, plasma, and corpus callosum by multiplex assay (Fig. 3). Interestingly, IL-1β was increased in the lung and corpus callosum, but not in the plasma of animals ventilated with high Vt (Fig. 3). Other cytokines assessed showed no difference among groups (Additional file 1: Table S3).

**Fig. 4:**
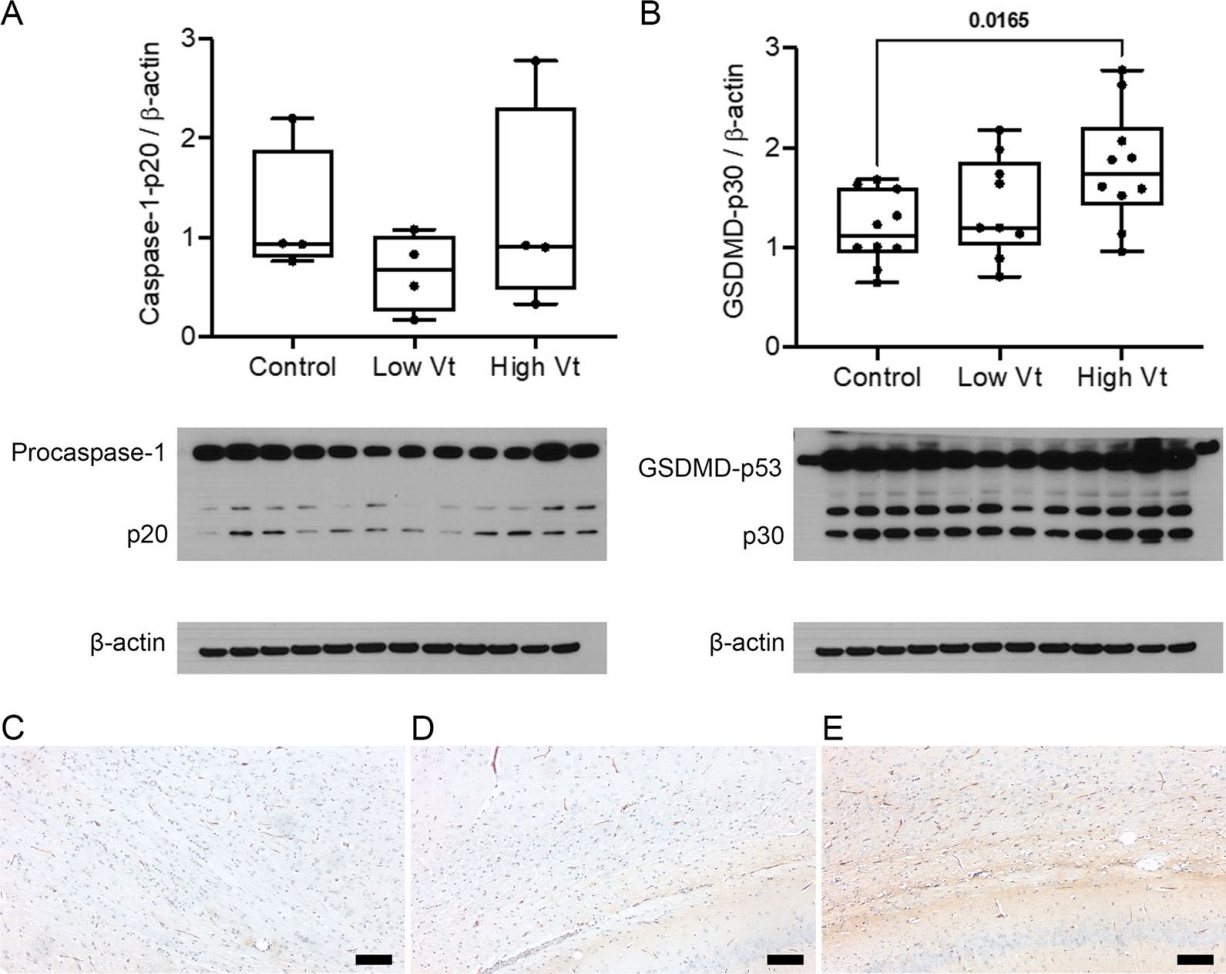
Injurious mechanical ventilation activated the pyroptosis effector GSDMD.** A**, **B** Western blot of the neonatal rat brain for caspase-1 (**A**) and GSDMD (**B**) showing increased relative band intensity of the active GSDMD fragment in whole brain homogenates from animals ventilated with high Vt. *N* = 4 animals/group (caspase-1) and *N* = 10 animals/group (GSDMD). Data was analyzed by ANOVA with Dunnett’s post-test, *p* values < 0.05 are shown on bars. Data are presented as box plots showing the median, 25th to 75th percentile, and minimal to maximal value normalized to control. **C–E** Photomicrographs (20×; bars = 50 µm) of representative brain sections immunostained for the GSDMD showing the corpus callosum and subcortical white matter in controls (**C**), low Vt (**D**), and high Vt (**E**) animals. There was increased staining for GSDMD in the corpus callosum and subcortical white matter of animals ventilated with high Vt. *N* = 4 animals/group. Data was analyzed by ANOVA with Dunnett’s post-test for multiple comparison, *p* values < 0.05 are shown on bars

As caspase-1 cleaves both pro-IL-1β and pro-GSDMD, we assessed the expression and activation of these proteins in the brain after mechanical ventilation (Fig. 4). High Vt induced activation of GSDMD (Fig. 4B) but not caspase-1 (Fig. 4A) in the brain. The increased expression of GSDMD localized to the corpus callosum and subcortical white matter by immunohistochemistry (Figs. 4C–E), suggesting that VILI induces GSDMD expression and activation in the neonatal white matter.

### Mechanical ventilation increases caspase-1 in circulating EVs

**Fig. 5:**
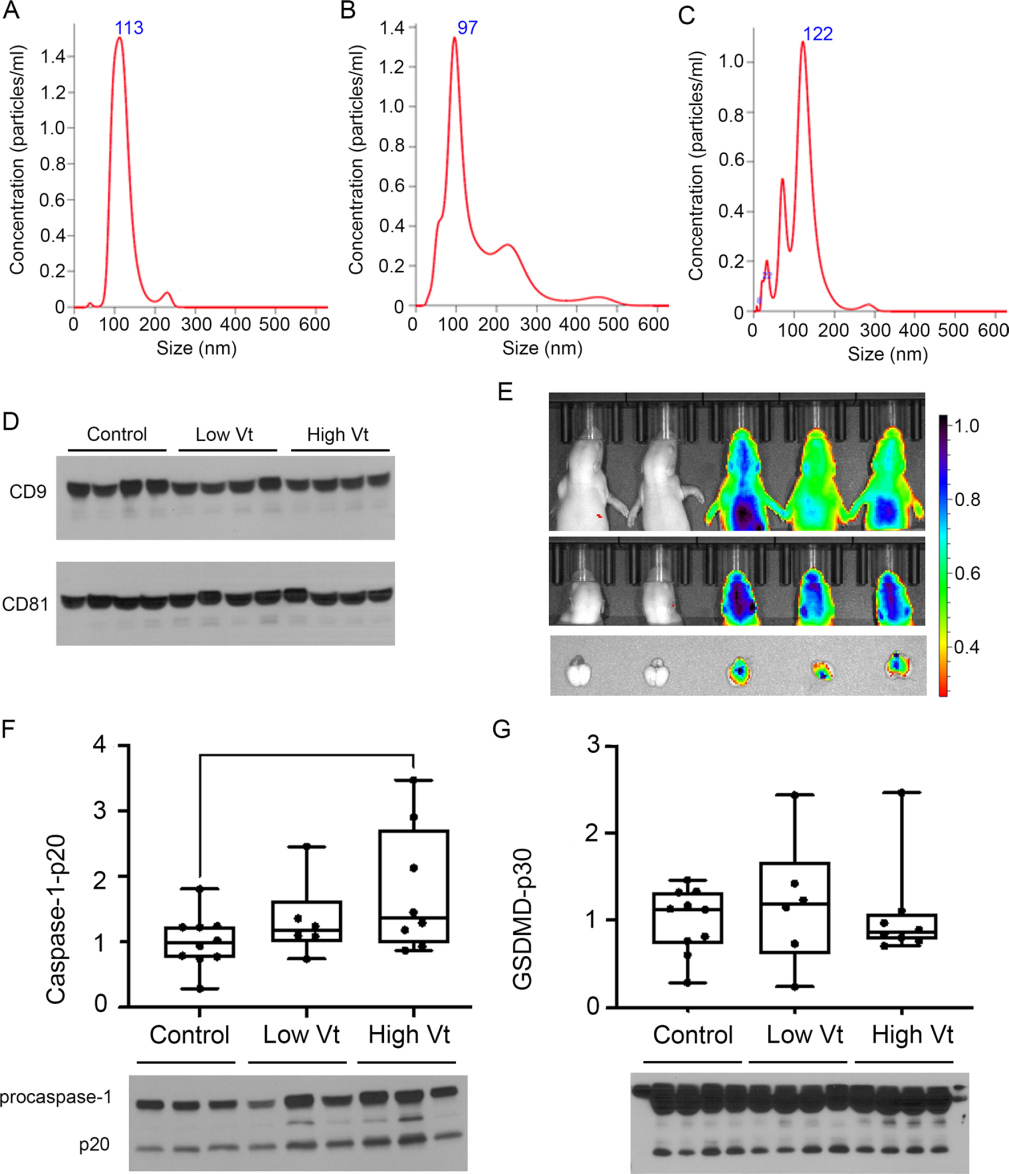
Circulating EVs from newborn rats with VILI are selectively loaded with caspase-1 and can cross the blood brain barrier. Nanoparticle tracking analysis of EVs isolated from controls (**A**), low Vt (**B**), and high Vt (**C**) show a distinct peak between around 100–120 nm, which corresponds to the exosome particle size. These EVs were enriched for the exosome markers CD9 and CD81 (**D**). Adoptive transfer by tail vein injection of fluorescence-labeled EVs shows that these EVs can cross the blood brain barrier and localize to the brain on in vivo and ex vivo imaging (**E**). Western blot of EVs extracted from controls and ventilated animals shows increased activated caspase-1 (**F**) but not GSDMD (**G**) in the EVs from animals with ventilation induced lung injury. Data are presented as box plots showing the median, 25th to 75th percentile, and minimal to maximal value normalized to control. *N* = 4 animals/group for nanoparticle tracking analysis. *N* = 10 controls, 6 low Vt, and 8 high Vt for EV Western blot analysis. Data was analyzed by ANOVA with Dunnett’s post-test for multiple comparison, *p* values < 0.05 are shown on bars

We hypothesized that circulating EVs could be mediating the lung–brain inflammation communication in rats with VILI. EVs were isolated from the plasma of controls and ventilated animals and analyzed by nanoparticle tracking (Nanosight), which revealed various particle size peaks ranging from 8 to 453 nm with a distinct exosome-sized particle peak at 90–120 nm, containing the majority of the EVs (Fig. 5A–C). Western blot for the exosome markers CD9 and CD81 also showed that isolated circulating EVs contained high levels of these markers (Fig. 5D). Given their size and lipid membranes, EVs have the ability to easily cross tissue barriers. To assess if the EVs isolated from the plasma could cross the blood brain barrier, they were tagged with the ExoGlow system and adoptively transferred via tail vein injection into normal newborn rats on P7 (Fig. 5E). In vivo imaging at 1 and 4 h showed that the EVs distributed through the body, including chest and head. At 4 h, these newborn rats were euthanized and their brains dissected. Ex vivo imaging showed the presence of the tagged EVs in the brain of these animals, proving that adoptively transferred EVs can cross the blood–brain barrier.

We hypothesized that the circulating EVs from animals with VILI would contain increased caspase-1 and GSDMD as we had observed in the lung. Western blot of these EVs for caspase-1 and GSDMD showed that the circulating EVs from rats with VILI had increased caspase-1 (Fig. 5E) but not increased GSDMD (Fig. 5F) compared to controls, suggesting that caspase-1 is selectively loaded into the EVs from ventilation-injured lungs.

### Adoptively transferred EVs can activate GSDMD in the brain

**Fig. 6:**
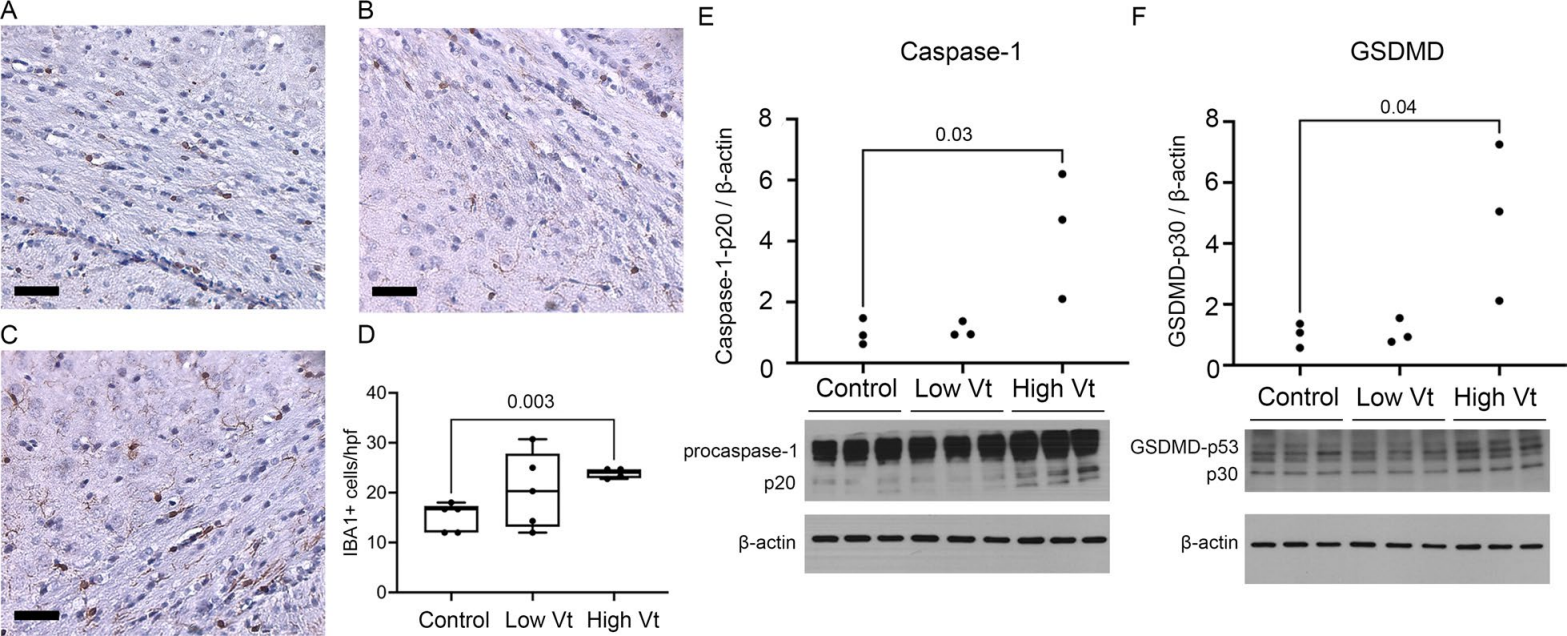
Adoptive transfer of circulating EVs from animals with ventilation-induced lung causes brain inflammation. IBA1 staining on brain sections of the corpus callosum and subcortical white matter of normal newborn rats who received adoptive transfer of circulating EVs from controls (**A**), low Vt (**B**), and high Vt (**C**) shows increased microglial count with exosomes from animals with VILI (**D**). Circulating EVs from animals with VILI also activated caspase-1 (**E**) and GSDMD (**F**) in the brain compared to exosomes from control animals. *N* = 5 controls, 5 low Vt, and 4 high Vt for IBA1 immunostaining. *N* = 3/group for caspase-1 and GSDMD western blot. Data is shown as box plots or individual data points. Western blot data is normalized to control. Data was analyzed by ANOVA with Dunnett’s post-test for multiple comparison, *p* values < 0.05 are shown on bars

To assess if circulating EVs from animals with VILI could activate GSDMD and induce cell death in the brain, we performed adoptive transfer of isolated EVs from controls, low Vt, and high Vt ventilated animals into normal newborn rats via tail vein injection on P7. We found that 2 weeks after adoptive transfer, EVs from animals with VILI caused neuroinflammation with increased microglial count in the corpus callosum (Fig. 6A–D) and increased expression of both active caspase-1 (Fig. 6E) and active GSDMD (Fig. 6F) in the brain. While there was an increase in GSDMD, there was no difference in the number of TUNEL positive cells (data not shown), suggesting that the inflammation and activation of GSDMD were not sufficient to induce pyroptosis.

## Discussion

The majority of extreme preterm newborns will require positive pressure ventilation for survival [17, 18]. Given the structural and biochemical immaturity of their lungs they are susceptible to VILI even after short periods of inadvertent high tidal volume delivery during neonatal resuscitation [19]. Lung injury from mechanical ventilation contributes to the development of bronchopulmonary dysplasia [20, 21]. Clinically, mechanical ventilation and bronchopulmonary dysplasia are associated with an increased risk of white matter injury and poor neurodevelopmental outcomes [1, 22], while a more restricted use of mechanical ventilation in preterm newborns is associated with improved neurodevelopmental outcomes [23]. With the increasing survival of periviable preterm newborns with more immature lungs, the use of mechanical ventilation will continue to be necessary in the management of preterm infants [24, 25]. Thus, understanding the relationship between VILI and brain injury is critical if we are to prevent brain injury and neurological impairments in preterm infants. Here, we report that circulating extracellular vesicles from newborn rats with ventilation-induced lung injury can cause inflammation and activation of pyroptosis mediators in the white matter when transferred into normal newborn rats. Our findings suggest a new injury signaling pathway in preterm newborns with lung injury.

While clinical studies speculate of a causal relationship between mechanical ventilation and brain injury [2], large animal models have provided insight into a causal relationship between mechanical ventilation and lung inflammation and injury [5]. In mechanically ventilated preterm lambs, mechanical ventilation is associated with acute microglial activation, oxidative stress, and caspase-mediated cell death [26–28]. RNA-seq from preterm lambs subjected to injurious mechanical ventilation for 15 min followed by 6 h of mechanical ventilation showed activation of inflammatory pathways, including IL-1β signaling and regulation of apoptosis pathways [29]. We have found an increase in IL-1β concentration in the corpus callosum, as well as microglial activation and increased cell death in the brain of animals with VILI compared to unventilated animals. In animals ventilated with a normal tidal volume we observed a non-significant trend of increased microglia but with similar IL-1β and apoptosis compared to unventilated animals suggesting there may be more subtle effects even with careful mechanical ventilation.

Circulating cytokines released from the injured lung are potential mediators of brain injury associated with mechanical ventilation. Two hours after initiation of mechanical ventilation there is an increased plasma concentrations of IL-1β, TNF-α, IL-8, IL-10, and IL-6 in preterm infants [30]. In addition, chronic mechanical ventilation for 14 days compared to less than 7 days is associated with even greater elevation of similar cytokines in the plasma [31]. Moreover, it is not clear how long the cytokine profile of infants with lung injury from mechanical ventilation remain elevated after positive pressure ventilation has been terminated. We found increased IL-1β in the lung and corpus callosum, but not in the plasma, suggesting that IL-1β is being endogenously produced in these organs. IL-1β is produced upon cleavage of the pro-IL-1 by caspase-1 [32]. Caspase-1 also cleaves GSDMD leading to cell membrane pore formation and pyroptosis [33]. We found increased activation of caspase-1 in the lung but not in brain of animals with ventilation-induced lung injury, while there was increased IL-1β and activated GSDMD both in the lungs and brain of these animals, with increased cell death assessed by TUNEL assay, suggesting activation of the pyroptosis in the brain after injurious mechanical ventilation. We hypothesized that EVs could be transporting caspase-1 from the injured lung into the brain, triggering inflammation and pyroptosis.

EVs have been implicated in physiological and pathological communication between cells inside the same organ and between distant organs [9, 13]. EVs can be classified according to their size, production mechanisms, and contents [34]. Although the plasma EVs isolated in our study were enriched for exosome markers and had nanoparticle tracking showed a peak size distribution within the range of exosomes, the tetraspanins CD9 and CD81 are not exclusive to exosomes and there were other peaks on nanoparticle analysis suggesting we had a mixture of different EVs in our isolates. Interestingly, the EVs isolated from animals with VILI had increased active caspase-1 similar to the lungs from these animals but not GSDMD. Caspase-1 has been directly implicated in the control of exosome release and pharmacological inhibition of caspase-1 decreases secretion of exosomes [35, 36]. Caspase-1 has also been implicated in the secretion of the inflammatory EVs that locally propagate the inflammatory response [37, 38]. The selective presence of caspase-1 but not GSDMD in the isolated EVs could be due to selective loading of caspase-1 into the EVs or due to the smaller increase of GSDMD relative to controls in the lung from ventilated animals. Alternatively, these circulating EVs may be coming from organs other than the lung.

Adoptive transfer of EVs from animals with VILI into normal animals induced activation of microglia and increased active caspase-1 and GSMD in the brain. However, the increased GSDMD was not sufficient to lead to increased cell death assessed by TUNEL assay. The lack of increased cell death in the presence of increased active GSDMD could be due to insufficient activation to trigger apoptosis or interaction of other factors influencing the execution of pyroptosis following activation of GSDMD, such as prevention of transition from the sublytic to lytic phase of pyroptosis [39] or removal of gasdermin pores by ESCRTIII [40]. One limitation of this study is that early increases in cytokines may be mediating the effects of mechanical ventilation on brain injury and this initial early increase in cytokines may be mediated by infiltrating peripheral leukocytes which was not assessed in this study. Another limitation of this study is the fact that some of the effects may be due to circulating cytokines isolated with the exosomes although there were no significant differences in cytokine concentrations in the plasma at this timepoint, except for a trend towards increased IL-6 in a few of the animals ventilated with high tidal volume (Additional file 1: Table S3).

## Conclusion

We show that, in newborn rats with VILI, circulating exosomes containing caspase-1 can cross the blood brain barrier and induce neuroinflammation and GSDMD activation. This pathway may contribute to the brain injury and poor neurodevelopmental outcomes seen in infants receiving injurious or prolonged mechanical ventilation and is a potential target for prevention of brain injury associated with mechanical ventilation in extremely preterm newborns.

## Supplementary Information

**Additional file 1: Table S1**. Primary antibodies’ information and dilutions used. **Table S2**. Secondary antibodies’ information and dilutions used. **Table S3**. Multiplex ELISA of lung, plasma, and corpus callosum of neonatal rats.

## Data Availability

The data sets used in this study are available from the corresponding author on reasonable request.

## Abbreviations

BSA: Bovine serum albumin
CD: Cluster differentiation
ECL: Enhanced chemiluminescence
ESCRTIII: Endosomal sorting complex required for transport 3
EV: Extracellular vesicle
GSDMD: Gasdermin D
HPF: High power field
HRP: Horseradish peroxidase
IBA1: Ionized calcium binding adaptor molecule 1
IL: Interleukin
P: Postnatal day
PBS: Phosphate buffer saline
TNF-α: Tumor necrosis factor alpha
TUNEL: Deoxyuridine triphosphate nick end labeling
VILI: Ventilation-induced lung injury
Vt: Tidal volume
