# Supplementary material for: Circulating extracellular vesicles activate the pyroptosis pathway in the brain following ventilation-induced lung injury
Source: J Neuroinflammation. 2021 Dec 29;18:310. doi: 10.1186/s12974-021-02364-z (PMC8717639; doi:10.1186/s12974-021-02364-z)
Supplement: Supplementary file 1 — Additional file 1: Table S1. Primary antibodies’ information and dilutions used. Table S2. Secondary antibodies’ information and dilutions used. Table S3. Multiplex ELISA of lung, plasma, and corpus callosum of neonatal rats. [file 12974_2021_2364_MOESM1_ESM.docx]

Supplemental table 1. Primary antibodies’ information and dilutions used.

| Target protein | Host | Manufacturer | Catalog | Application | Dilution |
| --- | --- | --- | --- | --- | --- |
| Beta-actin | Mouse | Sigma-Aldrich | A5441 | Western blotting | 1:10000 |
| Caspase-1 | Rabbit | Novus Biologicals | NB100-56565 | Western blotting | 1:500 |
| CD81 | Mouse | ThermoFisher | MA5-13548 | Western blotting | 1:500 |
| CD9 | Mouse | Invitrogen | 10626D | Western blotting | 1:500 |
| GSDMD | Rabbit | Sigma-Aldrich | G7422 | Western blotting | 1:5000 |
|  |  |  |  | Immunohistochemistry | 1:300 |
| IBA1 | Goat | Novus Biologicals | NB100-1028 | Immunohistochemistry | 1:500 |

Supplemental table 2. Secondary antibodies’ information and dilutions used.

| Reactivity | Host | Manufacturer | Catalog | Application | Dilution |
| --- | --- | --- | --- | --- | --- |
| Anti-mouse IgG | Goat | ThermoFisher | 31430 | Western blotting: caspase-1, beta-actin, CD9 | 1:10000 |
| Anti-rabbit IgG | Goat | ThermoFisher | 31460 | Western blotting: GSDMD | 1:5000 |
| Anti-goat IgG | Horse | Vector Labs. | BA9500 | Immunohistochemistry: IBA1 | 1:200 |
| Anti-rabbit IgG | Goat | Vector Labs. | BA1000 | Immunohistochemistry: GSDMD | 1:200 |

|  | Detection threshold  (pg/mL) | | Lung | | | Plasma | | | Brain | | |
| --- | --- | --- | --- | --- | --- | --- | --- | --- | --- | --- | --- |
|  | *Tissue* | *Plasma* | Control | Low Vt | High Vt | Control | Low Vt | High Vt | Control | Low Vt | High Vt |
| G-CSF | *1.22* | *18.83* | 2.9±0.4 | 2.8±0.8 | 3.2±1 | 44±7 | 58±4 | 69±34 | 7.4±3.3 | 6.9±1.0 | 5.0±0.9 |
| Eotaxin | *4.91* | *4.95* | 11±0.5 | 11±0.4 | 11±0.2 | 21±3 | 18±4 | 21±4 | 8.2±1.4 | 7.7±0.3 | 7.3±0.6 |
| GM-CSF | *3.05* | *93.04* | 19±7 | 19±5 | 20±8 | 79±57 | 35±16 | 98±43 | 30±17 | 32±9 | 28±23 |
| IL-1α | *27.58* | *12.21* | 176±20 | 203±19 | 189±22 | 57±32 | 49±36 | 81±62 | 33±11 | 31±8 | 32±8 |
| Leptin | *27.58* | *14.65* | 592±103 | 615 ±134 | 658±58 | 35102±  11715 | 35407±  7325 | 29462±  15803 | 135±32 | 133±4 | 148±22 |
| MIP-1α | *3.08* | *5.14* | 18±3 | 20±4 | 17±2 | 17±8 | 14±9 | 22±8 | 3.7±0.9 | 4.1±0.4 | 3.6±0.7 |
| IL-4 | *3.78* | *37.6* | 28±3 | 29±3 | 27±4 | 63±30 | 38±20 | 64±40 | 18±5.4 | 16±4 | 15±6 |
| IL-1β | *2.44* | *2.15* | 280±38 | 356±21 | 376±17 | 106±87 | 65±31 | 42±23 | 105±15 | 107±6 | 121±16 |
| IL-2 | *12.21* | *48.83* | 38±6 | 45±11 | 32±5 | 82±30 | 115±24 | 161±56 | 82±48 | 81±38 | 49±15 |
| IL-6 | *293* | *595* | 605±99 | 594±92 | 588±83 | 596 | 596 | 1346±  706 | 631±227 | 594±94 | 484±4 |
| EGF | *0.22* | *0.7* | 5.6±7.5 | 0.7±0.1 | 0.9±0.2 | <OOR | <OOR | <OOR | 0.4±01 | 0.3±0.03 | 1.4±2.3 |
| IL-13 | *4.88* | *6.14* | 18±3 | 15±2 | 16±1 | 43±17 | 31±20 | 49±38 | 28±12 | 24±7 | 17±1.4 |
| IL-10 | *1.83* | *6.96* | 48±7 | 51±3 | 54±6 | 170±150 | 100±63 | 86±21 | 79±13 | 74±12 | 96±24 |
| IL-12p70 | *12.21* | *40.3* | 18±5 | 21±4 | 20±4 | 158±110 | 167±20 | 197±21 | 18±3 | 17±3 | 17±46 |
| IFNγ | *41.26* | *181.48* | 278±39 | 294±14 | 296±42 | 363±173 | 486±12 | 458±81 | 284±69 | 303±25 | 269±12 |
| IL-5 | *18.5* | *6.78* | 31±6 | 28±2 | 30±5 | 102±25 | 116±33 | 138±20 | 65±32 | 52±46 | 43±60 |
| IL-17a | *1.85* | *5.57* | 9±0.9 | 9.5±1.5 | 11±3 | 42±15 | 44±13 | 57±24 | 90±50 | 96±22 | 50±39 |
| IL-18 | *3.05* | *7.23* | 4599±533 | 4692±556 | 4761±821 | 267±73 | 223±33 | 337±41 | 612±119 | 599±62 | 529±3 |
| MCP-1 | *117.19* | *117.19* | 108±22 | 111 ±46 | 131±24 | 1186±  232 | 1033±  608 | 1260±  357 | 155±42 | 154±57 | 135±20 |
| IP-10 | *0.44* | *0.93* | 55±4.3 | 60±6 | 61±9 | 314±70 | 253±29 | 271±44 | 35±5 | 38±4 | 39±12 |
| GRO/KC | *101.23* | *119.44* | 51±23 | 92±54 | 104±21 | 396±217 | 289±0 | 309±119 | 89±58 | 89±51 | 69±19 |
| VEGF | *1.11* | *8.80* | 4394±427 | 4481±272 | 4139±559 | 165±15 | 164±36 | 176±51 | 63±10 | 70±7 | 76±12 |
| Fractalkine | *2.44* | *4.22* | 234±30 | 264 ±68 | 255±51 | 209±41 | 217±26 | 218±50 | 824±233 | 741±368 | 1019±157 |
| LIX | *2.99* | *23.43* | 342±65 | 323±68 | 306±93 | 3659±  2753 | 1583±  139 | 2563±  2236 | 52±11 | 56±4.5 | 49±6 |
| MIP-2 | *26.89* | *16.81* | 30±1 | 42±10 | 33±5 | 27±14 | <OOR | 27±14 | 104±51 | 99±27 | 69±18 |
| TNF-α | *0.63* | *2.34* | 2.4±1.4 | 1.9±0.3 | 2.7±1.6 | 11±1.5 | 11±0.6 | 13±4.8 | 4.2±3 | 4.1±2.7 | 4.9±2.9 |
| RANTES | *1.25* | *1.29* | 509±141 | 560±232 | 528±200 | 859±  454 | 953±  744 | 1066±  905 | 5.7±0.9 | 5.5±1.7 | 6.7±1.1 |

Supplemental table 3. Multiplex ELISA of lung, plasma, and corpus callosum of neonatal rats.
